# 1758. Retrospective cohort analysis of outcomes in patients receiving outpatient parenteral antibiotic therapy (OPAT)

**DOI:** 10.1093/ofid/ofac492.1388

**Published:** 2022-12-15

**Authors:** Kaylyn Billmeyer, Jennifer Ross, Elizabeth B Hirsch, Michael Evans, Susan E Kline, Alison Galdys

**Affiliations:** University of Minnesota College of Pharmacy, Minneapolis, Minnesota; M Health Fairview - University of Minnesota Medical Center, Minneapolis, Minnesota; University of Minnesota College of Pharmacy, Minneapolis, Minnesota; University of Minnesota, Minneapolis, Minnesota; University of Minnesota Medical School, Minneapolis, Minnesota; University of Minnesota, Minneapolis, Minnesota

## Abstract

**Background:**

While many infectious conditions can be safely treated with oral antimicrobials, select circumstances require parenteral antimicrobial therapy. The potent nature of agents administered through OPAT present an increased risk of adverse events that may require unscheduled medical care. We analyzed the outcomes among patients receiving OPAT as part of the implementation of a collaborative OPAT program.

**Methods:**

Adult patients discharged home from an academic hospital with OPAT between 1/2019 and 6/2021 were included in this retrospective cohort study. Patients with cystic fibrosis were excluded. Data on patient characteristics and outcomes was collected from electronic medical records by two reviewers using a standardized REDCap instrument. Multivariable analysis was conducted to identify predictors of vascular access device (VAD) complications, adverse drug events, (ADEs) unscheduled care, and clinical failure. Analyses were conducted with R versions 4.1.1 and 4.0.4 (R Foundation for Statistical Computing). IRB approval was received.

**Results:**

The cohort comprised 265 unique patients. The most common OPAT indication was bloodstream infection, and cephalosporins were the most frequently prescribed antimicrobial class. (Table 1) Fifty-six (21.1%) patients experienced a VAD complication, and 82 (30.9%) an ADE. Fifty-eight (21.9%) patients experienced an OPAT-related ED visit, 53 (20.0%) experienced an OPAT-related rehospitalization, and 15 (6.1%) experienced clinical failure.

Patients who received >1 antimicrobial agent were more likely to experience ADEs. VAD complications were more common in obese patients. (Figure 1).

VAD complication was associated with OPAT-related ED visits (OR 2.73, p=0.0067) and clinical failure during OPAT (OR 7.11, p=0.0012). ADE was associated with 90-day rehospitalization (OR 2.59, p=0.0067). Skin/soft tissue, genitourinary, and bloodstream infections were associated with less unscheduled care and clinical failure. (Table 2).

**Table 1.**
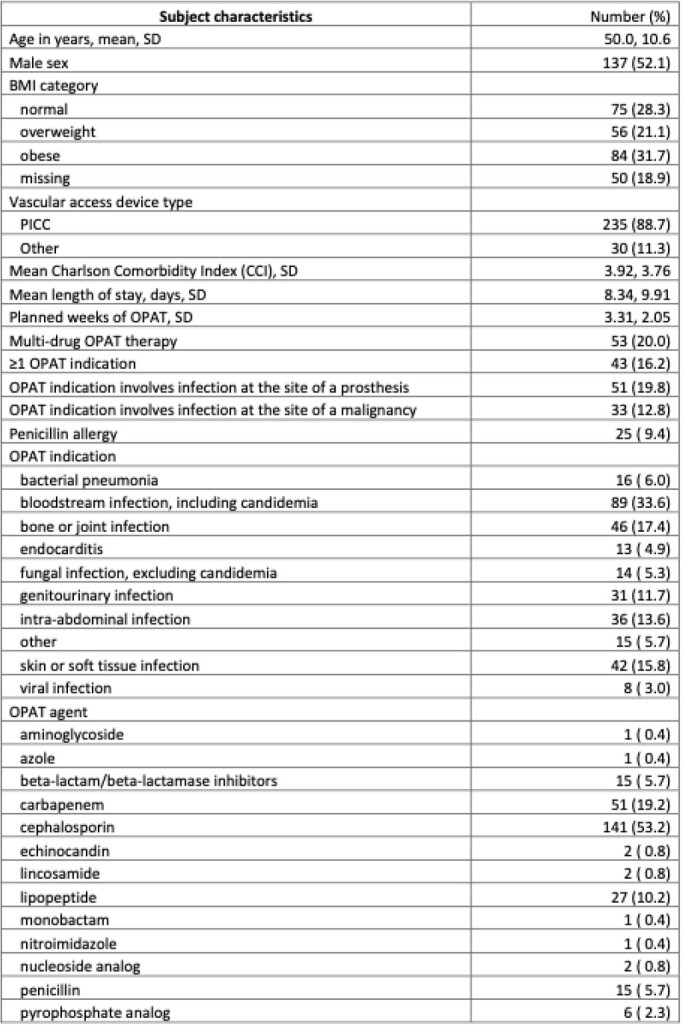
Characteristics and outcomes of study population of 265 patients, all of whom received outpatient parenteral antibiotic therapy (OPAT)

**Figure 1.**
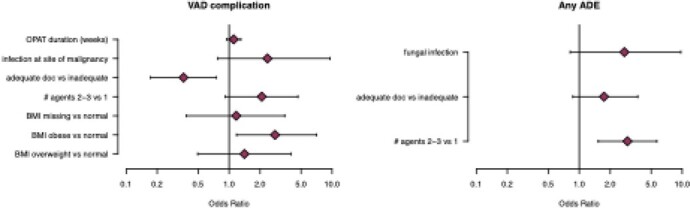
Multivariable logistic regression analyses (OR and 95% CI) for vascular access device (VAD) complications and adverse drug events (ADEs). Adequate versus inadequate documentation of OPAT plan was determined by reviewers.

**Table 2.**
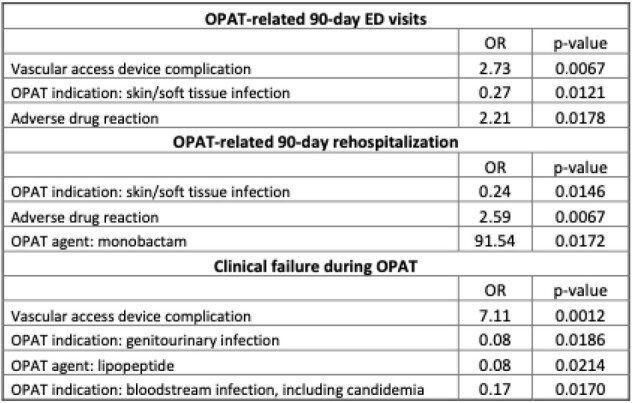
Multivariable logistic regression analysis of factors associated with OPAT-related emergency department (ED) visits, rehospitalizations, and clinical failure.

**Conclusion:**

OPAT-related unscheduled care was frequent in our cohort, especially among patients who experienced VAD complications or adverse drug events. Antimicrobial selection and duration should be optimized to reduce OPAT-related adverse events.

**Disclosures:**

**Elizabeth B. Hirsch, PharmD, FCCP, FIDSA**, Melinta: Advisor/Consultant|MeMed: Advisor/Consultant|Merck: Advisor/Consultant|Merck: Grant/Research Support.

